# Comparative efficacy of therapeutic modalities for metastatic uveal melanoma: a systemic review and network meta-analysis

**DOI:** 10.3389/fonc.2026.1811719

**Published:** 2026-05-20

**Authors:** Qinyun Chen, Jie Zhou, Hongli Zhou, Hao Wang, Jianhua Li

**Affiliations:** 1 Department of Ophthalmology, Affiliated Hospital of North Sichuan Medical College, Nanchong, Sichuan, China; 2 Department of Ophthalmology, Chengdu Second People’s Hospital, Chengdu, Sichuan, China; 3 Department of Ophthalmology, Chongqing University Fuling Hospital, Chongqing, China

**Keywords:** metastatic uveal melanoma, immunotherapy, liver-directed therapy, tebentafusp, network meta-analysis

## Abstract

**Objective:**

To evaluate the efficacy of different therapeutic modalities in the treatment of metastatic uveal melanoma (mUM).

**Methods:**

According to PRISMA criteria, We identified relevant randomized controlled trials (RCTs) by searching PubMed, Embase, and The Cochrane Library through March 31, 2026. Patients with liver metastatic uveal melanoma were enrolled. The analysis of clinical prognostic factors was performed using R 4.2.0. The main outcomes measured were overall survival (OS) and progression-free survival (PFS).

**Results:**

A total of 16 articles were screened between 2000 and 2026, involving 2585 patients. The trials evaluated eight treatment approaches: tebentafusp, immune checkpoint inhibitors (ICIs), targeted therapy, targeted therapy plus chemotherapy, chemotherapy, liver-directed therapy (LDT), liver-directed therapy combined with ICIs, and liver-directed therapy plus chemotherapy. The results of the included trials showed that in terms of overall survival and progression-free survival, the liver-directed therapy combined with ICIs were the most effective regardless of the HLA genotype. Tebentafusp showed the second-best OS but the worst PFS among the compared treatments. Immune checkpoint inhibitors were inferior to tebentafusp in improving OS but were superior in PFS. Furthermore, compared with conventional systemic chemotherapy, targeted therapy, or their combination, regional liver-directed therapy demonstrated more favorable outcomes in both OS and PFS. Emerging immunotherapies (e.g., tumor vaccines, oncolytic virotherapy, tumor-infiltrating lymphocytes) and novel targeted agents could not be included in the NMA due to the absence of comparative trials or ongoing investigations.

**Conclusion:**

The liver-directed therapy combined with ICIs achieved the best results compared to Tebentafusp, ICIs and other therapeutic modality for OS and PFS extension in metastatic uveal melanoma based on available data. Future comparative studies incorporating emerging therapies are warranted.

**Systematic Review Registration:**

https://www.crd.york.ac.uk/prospero/, identifier CRD420261393862.

## Introduction

Uveal melanoma represents the most frequent primary intraocular malignancy in the adult population, with an annual incidence of approximately 6 cases per million individuals among Caucasian populations. It accounts for around 3% of all melanoma diagnoses (1). Although primary tumor control is often achievable, this disease carries a substantial risk of systemic dissemination, with metastases developing in nearly 50% of patients. The liver is the predominant site of metastatic spread, involved in up to 90% of cases. Prognosis following metastatic relapse remains poor, with a median survival of approximately 12 months. Survival rates decline sharply over time—from 52% at one year to 25% at two years and just 13% at three years (2, 3).

Survival outcomes in metastatic uveal melanoma (mUM) have remained poor, primarily due to the limited efficacy of available therapeutic options. Conventional chemotherapy demonstrates limited efficacy in the management of metastatic uveal melanoma and fails to improve survival rates (4, 5). While some retrospective studies indicate potential benefit of immune checkpoint inhibitors (ICIs) (6, 7), treatment with ipilimumab or nivolumab, either alone or in combination, yields low objective response rates of 10% to 18% and an uncertain survival benefit (8, 9).

Encouragingly, a recent phase III trial revealed a survival benefit associated with tebentafusp—a bispecific T-cell engager—in patients with mUM, compared to chemotherapy or ICIs monotherapy. The median overall survival was 21.7 months in the tebentafusp group versus 16.0 months in the control group (hazard ratio for death, 0.51) (10). Tebentafusp is designed to redirect T cells to target cells expressing a peptide derived from glycoprotein 100, a melanoma-associated antigen, presented in the context of HLA-A*02:01. Although tebentafusp has gained approval from both the U.S. Food and Drug Administration and the European Medicines Agency as the first systemic therapy for mUM, its application is restricted to the approximately 50% of Caucasian individuals who carry the HLA-A*02:01 genotype (11). For those lacking this genotype, no clear standard of care currently exists.

Beyond above immunotherapies, tumor vaccines (12) and oncolytic virotherapy (13) are now under active development. Separately, the sustained clinical responses achieved with tumor-infiltrating lymphocyte (TIL) in advanced cutaneous melanoma present a viable translational approach for mUM (14).

Emerging therapeutic strategies for mUM include novel targeted therapy such as MEK inhibitors. Although agents like selumetinib and trametinib have shown initial efficacy in clinical trials but fail to improve overall survival owing to acquired resistance (15). Current research is exploring Phosphoinositide 3-kinase (PI3K) inhibitors as a promising strategy to overcome resistance to MEK-targeted therapies (16). Meanwhile, darovasertib (NVP-LXS196) — a potent pan-PKC inhibitor with high kinase-wide selectivity — has been identified and is currently under clinical investigation as monotherapy, with potential combinations being explored for uveal melanoma treatment (17). Additionally, the histone deacetylase (HDAC) inhibitor entinostat has been found to induce apoptosis and revert UM cells to a differentiated, melanocytic gene expression profile, and is now a key focus of research (18).

Management of mUM exhibits substantial geographical variation, influenced by disparities in local expertise and institutional practices, with no established consensus on therapy strategy. Given that liver-only or liver-predominant disease occurrence is common, researchers also consider focusing on the liver therapy management. The concept of liver-directed therapy (LDT) was first introduced in 1961 by Dr. Robert K. Ausman, marking a turning point in metastatic liver cancer treatment (19). A meta-analysis of trials published between 2000 and 2015 indicated that liver-directed regional therapies were associated with significantly improved progression-free survival (median PFS 5.2 months) and overall survival (median OS 14.6 months) compared to systemic therapy (median PFS 2.8 months, median OS 9.3 months), independent of prognostic characteristics (4).

The regional liver-directed therapies includes surgical resection (20), radiofrequency ablation (21), transarterial chemoembolization (TACE) (22), immunoembolization (23), selective internal radiotherapy (SIRT) (24), hepatic arterial infusion chemotherapy (HAIC) (25), localized delivery of chemotherapy by isolated hepatic perfusion (IHP) (26), and percutaneous hepatic perfusion (PHP) (27). Some of these treatments demonstrated superior efficacy compared to systemic therapy in phase III clinical trials (17).

Therefore, locoregional treatment, either as monotherapy or in combination with systemic therapy, should be considered for patients with liver-predominant metastatic uveal melanoma when clinically feasible. Given the limited response to immunotherapy in mUM, current investigative strategies focus on combining immunotherapy with other systemic or liver-directed treatments to enhance antitumor efficacy.

There is no standard of care available for liver metastatic uveal melanoma (LMUM) until now. Clinicians continue to use single-agent ICIs for mUM in real-world settings despite poor response rates, reflecting the limited global availability of tebentafusp. Heterogeneity in patient selection and treatment protocols precludes meaningful cross-modality comparisons. Existing efficacy data derive primarily from small observational studies and single-arm trials, limiting interpretability. Moreover, while emerging therapeutic strategies are rapidly evolving, their clinical data remain predominantly benchmarked against historical or conventional treatments. Direct comparisons between these newer modalities are scarce, leaving critical gaps in understanding their relative efficacy and optimal sequencing.

In this systematic review and network meta-analysis, we aim to quantify the comparative efficacy of diverse therapeutic modalities where head-to-head trials are absent, to inform clinical decision-making and address current therapeutic uncertainties. By evaluating available evidence, this study seeks to bridge the unmet need for efficacy comparisons in the state-of-the-art treatment strategies for mUM and discuss their role in the contemporary treatment paradigm of mUM.

## Methods

### Search strategy and selection criteria

This study was conducted in accordance with the PRISMA extension for network meta-analysis (28). Comprehensive literature searches were performed using PubMed, Embase, and the Cochrane Central Register of Controlled Trials to identify eligible studies. To minimize publication bias, additional searches included clinicaltrials.gov for unpublished trials, supplementary materials from relevant articles, and conference proceedings from major oncological meetings such as those of the American Society of Clinical Oncology (ASCO) and the European Society for Medical Oncology (ESMO). All relevant publications were performed from 2000 to March 31, 2026.

The search focused on the keywords “immunotherapy”, “immune checkpoint inhibitors”, “metastatic uveal melanoma”, “liver metastatic uveal melanoma”, “tebentafusp”, “targeted therapy”, and “liver-directed therapy.” The detailed search strategy is provided in the Supplementary Material (**Supplementary Table 1**).

Inclusion criteria for this study included: (a) patients with metastatic uveal melanoma (liver metastases of uveal melanoma); (b) trials comparing any two or more different groups of the following treatments: immunotherapy, immune checkpoint inhibitors, tebentafusp, chemotherapy, regional liver-directed therapies, targeted therapy; (C) outcome measures: included studies reporting one or more of the following outcomes: a. Progression-free survival, defined as the time from randomization to the first occurrence of disease progression (local or distant) or death; b. Overall survival, defined as the time from randomization to death from any cause; (D) randomized controlled trials (RCTs).

We excluded all studies that did not meet the inclusion criteria. Exclusion criteria comprised: (a) non RCTs, including meta-analyses, letters, case series, case reports, and reviews; (b) studies lacking clear baseline characteristics; (c) duplicate publications; (d) the comparison between the same type of treatment; (e) hazard ratios cannot be acquired.

All studies were reviewed independently by two investigators (HW and JZ). Differences of opinion were resolved by discussion or by a third researcher (HLZ) until a consensus was reached. The completed PRISMA checklist is provided in the Supplementary Material (**Supplementary Table 2**).

### Quality assessment and data extraction

Methodological quality of included studies was assessed independently by two investigators (HW and JZ) using Cochrane Risk of Bias tool. Evaluated domains covered random sequence generation, allocation concealment, blinding of participants and personnel, blinding of outcome assessment, incomplete outcome data, selective reporting, and other potential biases. Discrepancies were resolved through consensus or via consultation with a third reviewer. Primary endpoints comprised overall survival (OS) and progression-free survival (PFS), expressed as hazard ratios (HR) with 95% confidence intervals (CI). Where HRs were not directly reported, estimates were derived using established methods by Tierney et al (29). (2007) based on available aggregate data. Quality of non-randomized studies was evaluated using Newcastle-Ottawa Scale.

### Data synthesis and statistical analysis

The network meta-analysis was conducted within a Bayesian framework utilizing JAGS and the “gemtc” package in R software (version 4.0.2). Network diagrams were generated to illustrate comparative relationships among treatment regimens for different outcomes. Heterogeneity between studies was evaluated using forest plots and quantified with I² statistics, with values below 50% considered low and above 50% deemed high. Model selection was guided by I² and Deviance Information Criterion (DIC) values. Noninformative priors were specified, and posterior distributions were sampled using four Markov chains, each with 50,000 iterations following 20,000 burn-in steps and a thinning interval of 10. Results were reported as hazard ratios with 95% CI for each outcome. Additionally, ranking probabilities for each treatment regimen were computed to estimate the likelihood of being the optimal intervention. Rank probability plots were generated to visualize relative efficacy across all treatments. Statistical significance was defined by 95% CIs excluding the null value of 1.

## Results

### Study selection

**Figure 1:**
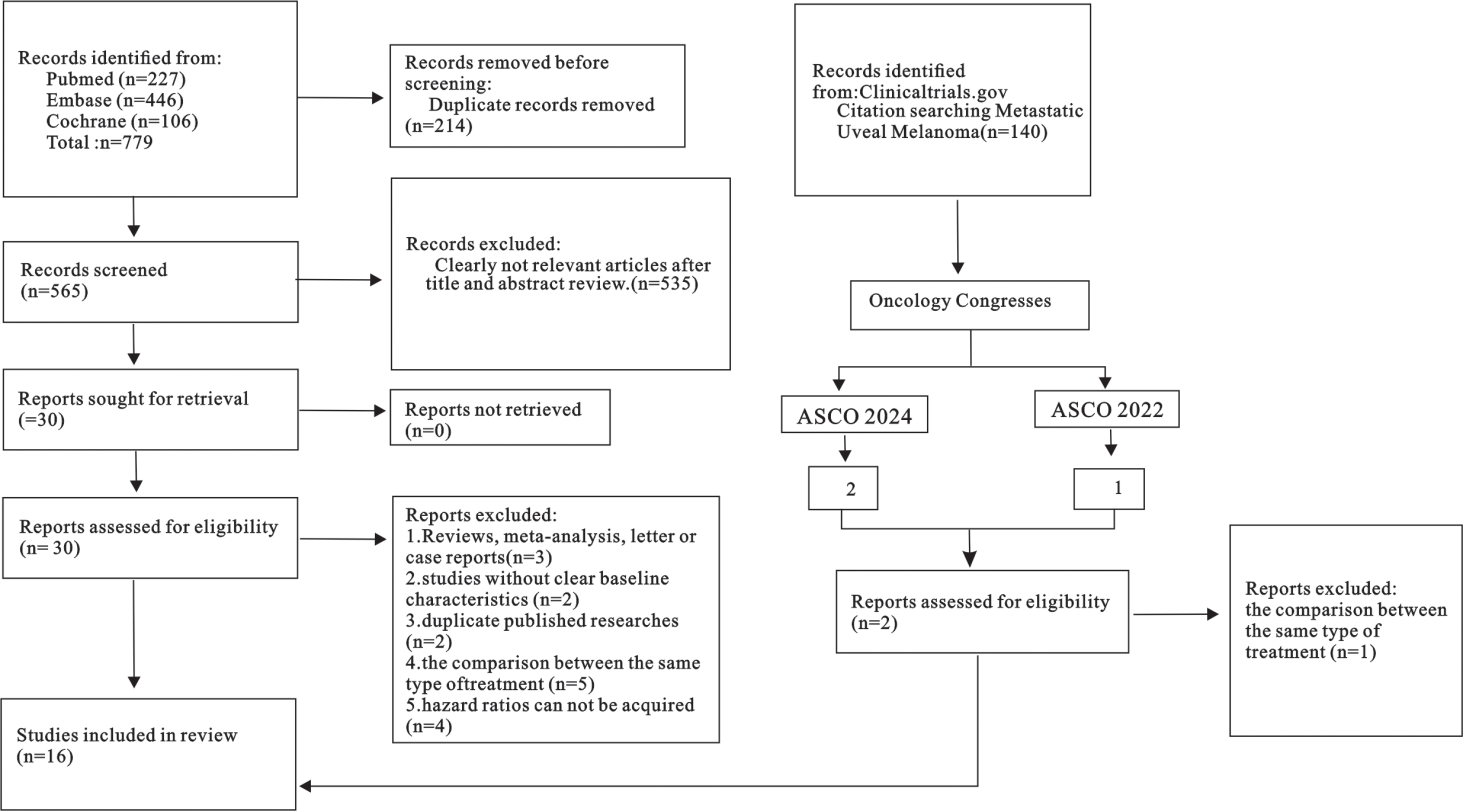
Flow diagram representing the selection process.

Based on the initial search, a total of 994 articles were identified as relevant. After eliminating 298 duplicates, an additional 664 articles were excluded based on the predetermined inclusion criteria. Subsequently, the complete texts of the remaining 32 studies that were potentially eligible underwent comprehensive screening. In the same time, 2 eligible research reports form the ASCO were included. As a result, 16 controlled trials were found to meet the criteria and were included in the final analysis (**Figure 1**).

### Study characteristics

This study analyzed a cohort of 2585 patients and investigated 8 different treatment approaches totally. Among the literature reviewed, there were 16 studies reporting the 17 outcomes of PFS and OS (10, 25, 26, 30–38), meanwhile 2 studies reporting the OS index only (39, 40). The evidence base comprised 9 randomized trials and 7 retrospective comparative studies. All participants were adult patients with a confirmed diagnosis of metastatic uveal melanoma particularly liver metastases. Specifically, 6 studies focused on patients with previously untreated metastatic uveal melanoma (10, 26, 30, 31, 34, 37). The treatment of immunotherapy mainly referred to immune checkpoint inhibitors including pembrolizumab, ipilimumab, and nivolumab. The targeted therapy contained series of kinase inhibitors, such as selumetinib, cabozantinib, sunitinib etc. The chemotherapy included dacarbazine, fotemustine and temozolomide. Liver-directed therapies such as SIRT, IHP and PHP, were applied to local treatment strategy. The most frequently used comparison regimen among the included studies were chemotherapy and liver-directed therapy (n = 4). The treatment protocols utilized in the 16 studies were as follows: tebentafusp in 1 study (10), ICIs in 4 studies (10, 30, 38, 39), targeted therapy + chemotherapy in 2 studies (31, 34), targeted therapy in 4 studies (31, 33, 35, 41), chemotherapy in 9 studies (25, 26, 34, 35, 37, 40, 42), liver-directed therapy combined with ICIS (LDT-ICIs) in 4 studies (32, 36, 38, 39), liver-directed therapy (LDT) in 7 studies (25, 26, 32, 36, 37, 40, 42), LDT plus chemotherapy in 1 study (40). A summary of the key characteristics of the included studies can be found in **Table 1**.

**Table 1:** Baseline characteristics of studies.

No	Study/year	N	E/C	Network comparator (s)	PFS, months	PFS,HR (95%CI)	OS, months	OS,HR (95%CI)	NOS score	ECOG score	The median follow-up months	Median age, years	Male %	ORR%	Grade 3 or higher adverse event	Time to first diagnosis of liver metastasis, months
1	Jessica C et al. (2024) (43)	373	247	tebentafusp	3.4	0.76 (0.6-0.97)	21.6	0.68 (0.54-0.87)	NA	≤1	43.3	64	51%	11%	47%	NA
			126	pembrolizumab/ipilimumab	2.9		16.9					66	49%	5%	18%	
2	Joseph J et al. (2024) (31)	77	51	selumetinib+paclitaxel	4.8	0.62 (0.41-0.92)	9	0.98 (0.58-1.66)	NA	≤2	11.2	64	54%	14%	53%	41.7
			26	selumetinib	3.4		10					64.5	46%	4%	77%	60.1
3	Toulsie et al. (2022) (30)	105	55	Pembrolizumab/nivolumab/ipilimumab	3.9	0.81 (0.5-1.33)	26.8	0.2 (0.09-0.45)	8	≤1	NA	60	51%	5%	NA	NA
			50	Dacarbazine/fotemustine	3.2		13.9					61	49%	2%		
4	Veronica et al. (2022) (32)	32	18	SIRT+ipilimumab+nivolumab	4.6	0.98(0.54-1.79)	49.6	0.4 (0.16-1.01)	8	≤1	23.9	61	33.30%	22.20%	44.40%	28.8
			14	SIRT	4.9		13.6					61	50%	14.30%	35.70%	21
5	Chiara L et al. (2021) (39)	42	19	LDT-ICIs	NR	NR	20.5	0.44(0.21-0.92)	7	≤1	NA	63	73.30%	NA	NA	20.4
			23	ICIs		NR	11.4					63	73.30%	NA	NA	20.4
6	Jason J et al. (2020) (33)	46	31	cabozantinib	2	0.99 (0.51- 1.86)	6.4	1.21(0.62- 2.34)	NA	≤1	25	60	54.80%	NA	51.60%	NA
			15	Temozolomide or dacarbazine	1.9		7.3					67	60.00%	NA	20.00%	
7	Richard D et al. (2018) (34)	129	97	Selumetinib+dacarbazine	2.8	0.78 (0.48- 1.27)	Not mentioned	0.75 (0.39- 1.46)	NA	≤1	NA	63	57%	3%	63%	NA
			32	placebo+dacarbazine	1.8							58	41%	0%	53%	
8	Richard D et al. (2014) (35)	101	50	selumetinib	3.7	0.46 (0.3- 0.71)	11.8	0.66 (0.41- 1.06)	NA	≤1	15	62	52%	14%	37%	NA
			51	temozolomide or dacarbazine	1.6		9.1					62	62%	0%	20%	
9	Rino S et al. (2024) (41)	74	38	Sunitinib	2.8	1.09 (0.62- 1.92)	6.4	1.59 (0.86- 2.96)		NA	NA	NA	NA	0%	NA	NA
			36	dacarbazine	3.9		8.7							8%		
10	Alexa O et al. (2019) (36)	24	12	Y-90 TARE plus immunotherapy	10.3	0.19 (0.07- 0.49)	26	0.38 (0.17- 0.84)	8	≤1	43	54	66%	8%	NA	NA
			12	Y-90 TARE	2.7		9.5					54	50%	10%	NA	
11	Marybeth S et al. (2015) (37)	93	44	PHP-MEL	5.4	0.404(0.252- 0.65)	10.6	0.92 (0.52 -1.62)	NA	≤1	NA	55	52.30%	27.30%	62%	NA
			49	Chemotherapy	1.6		10					56	44.90%	4.10%	NA	
12	Rino S et al. (2020) (40)	278	80	Chemotherapy			5.3	2.09 (1.21- 3.62)	7	NA	40.8	60	51%	NA	NA	35.9
			198	LDT			13.6				62.6	53	47%	NA	NA	41.7
13	Rino S et al. (2020) (40)	650	198	LDT			13.6	1.06 (0.61 -1.86)	7	NA	62.6	53	47%	NA	NA	41.7
			452	LDT plus chemotherapy			17.8				55.4	57	55%	NA	NA	35.6
14	Rogeret al. (2023) (26)	85	41	IHP	7.4	0.21(0.12 -0.36)	21.7	0.64 (0.37 -1.1)	NA	≤1	24	65	44%	NA	19.50%	20.6
			44	Chemotherapy	3.3		11.6					68	64%	NA	6.50%	29.7
15	Jonathan S et al. (2024) (42)	123	91	PHP-MEL	9.03	0.2 (0.12- 0.33)	20.53	0.63 (0.35- 1.12)	NA	NA	NA	NA	NA	35.20%	42.60%	NA
			32	Chemotherapy	3.12		14.06							12.50%	NA	
16	Elias A et al. (2023) (38)	182	78	LDT-ICIs	3	0.79 (0.46- 1.36)	20.1	0.62 (0.32- 1.21)	8	≤1	NA	63.8	53.80%	16.70%	52.60%	14.4
			104	ICIs	2.5		13.8			≤2		66.2	47.10%	3.90%	38.50%	8
17	Leyvraz et al. (2014) (25)	171	86	HIA fotemustine	4.5	0.62 (0.45 -0.84)	14.6	0.98 (0.71- 1.35)	NA	≤1	67.2	58	47%	10%	21.20%	NA
			85	chemotherapy	3.5		13.8					60	53%	2%	42.10%	

NA, Not Available.

### Therapeutic effect

#### Progression-free-survival

**Figure 2:**
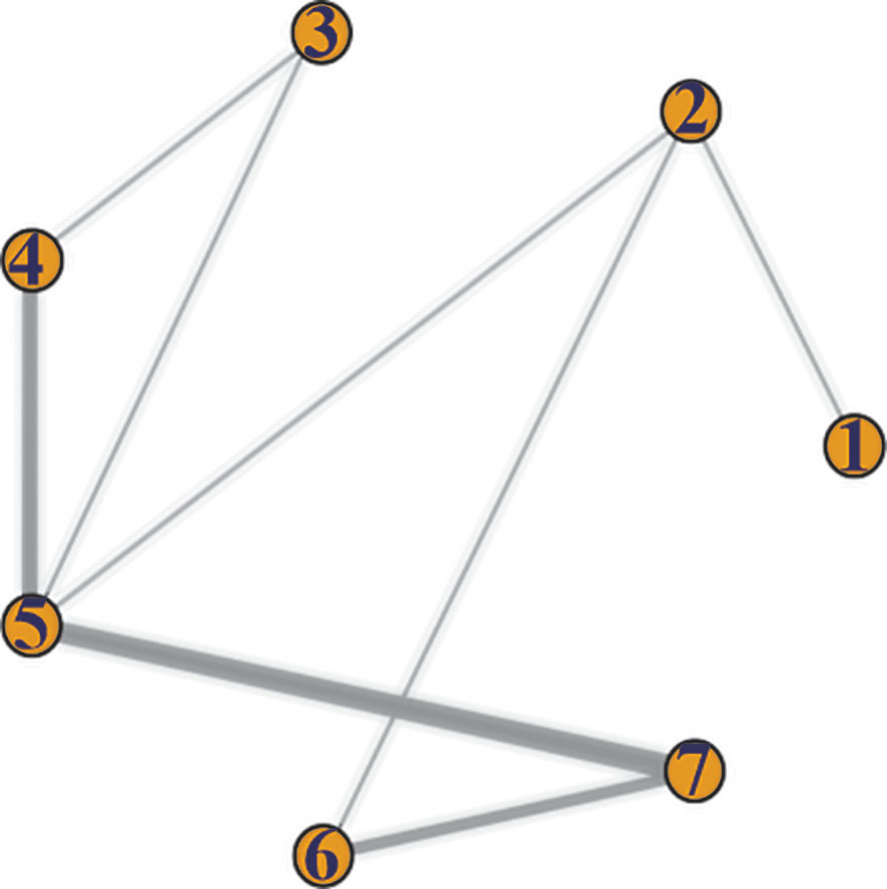
Network diagram of PFS. 1. tebentafusp; 2. ICIs; 3. targeted therapy combined with chemotherapy; 4. targeted therapy alone; 5. chemotherapy; 6. LDT-ICIs; 7. LDT.

A total of 15 studies reported the data of progression-free survival. The efficacy of 7 different treatment modalities was compared in above studies. Comparative network plots were illustrated in **Figure 2**.

**Figure 3:**
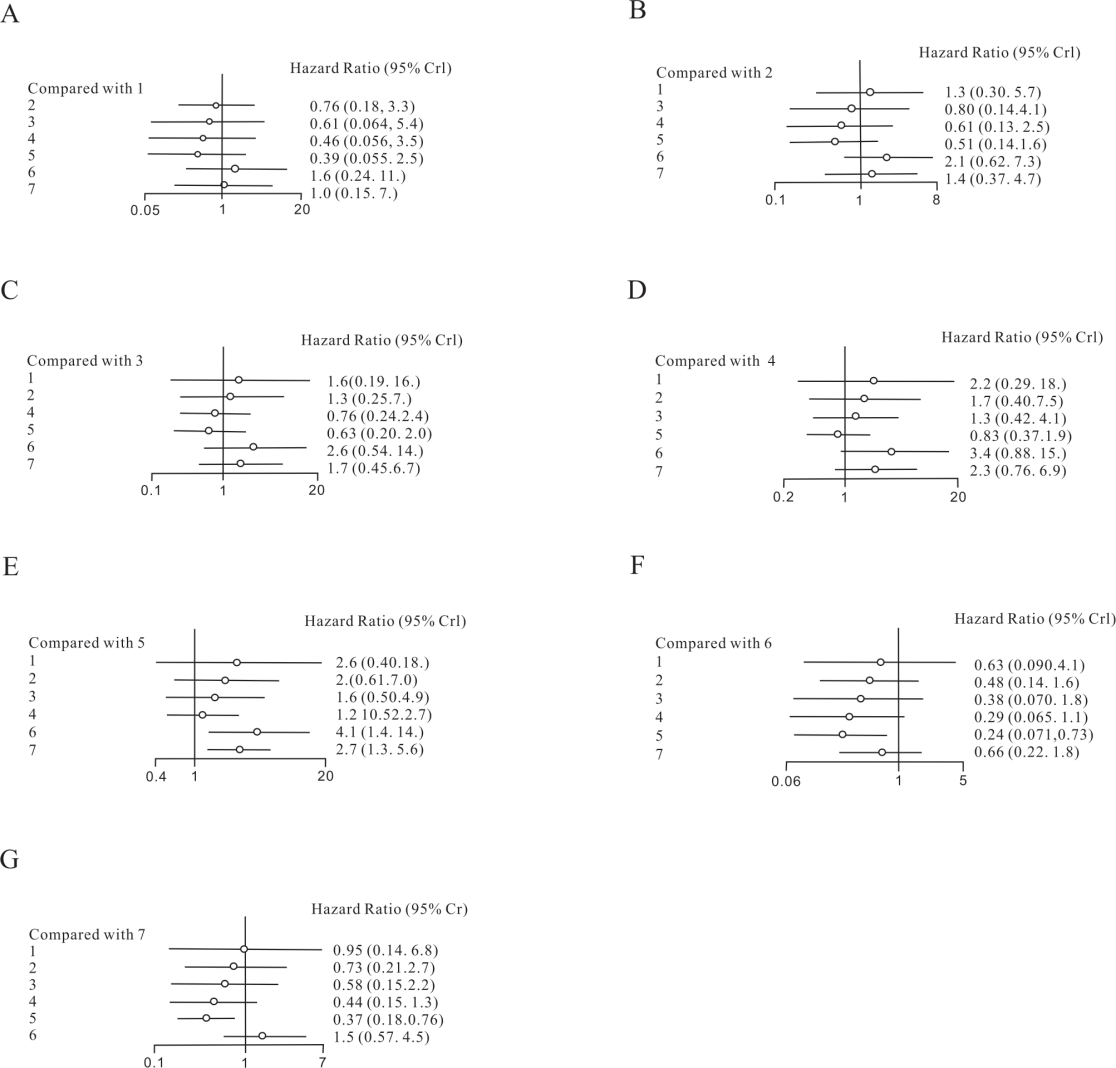
Forest plot of comparison of PFS. 1. tebentafusp; 2. ICIs; 3. targeted therapy combined with chemotherapy; 4. targeted therapy alone; 5. chemotherapy; 6. LDT-ICIs; 7. LDT. **(A)** compared with tebentafusp; **(B)** compared with ICIs; **(C)** compared with targeted therapy combined with chemotherapy; **(D)** compared with targeted therapy alone; **(E)** compared with chemotherapy; **(F)** compared with LDT-ICIs; **(G)** compared with LDT.

The forest plot for PFS (**Figure 3**) showed that only liver-directed therapy alone (HR 2.7, 95% CI: [1.3,5.6]) and LDT-ICIs (HR 4.1,95% CI: [1.4,14]) showed superiority to chemotherapy. In terms of ICIS, there were no statistically significant differences among tebentafusp (HR 1.3, 95% CI: [0.30,5.7]),targeted therapy plus chemotherapy (HR 0.8, 95% CI: [0.13,4.1]), targeted therapy alone (HR 0.61, 95% CI: [0.13,2.5]), chemotherapy (HR 0.51, 95% CI: [0.14,1.6]), LDT-ICIs (HR 2.1,95% CI: [0.62,7.3]), and LDT (HR 1.4,95% CI: [0.37,4.7]).

The relative effect rankings excel is illustrated in **Table 2**. The longest PFS achieves the highest position. The LDT-ICIS ranks first. Following closely behind are liver-directed therapy alone, ICIs, and targeted therapy plus chemotherapy. However, the chemotherapy and tebentafusp rank lower than targeted therapy. Unexpectedly, tebentafusp ranks last.

**Table 2:** Ranking chart of PFS.

Therapeutic modality	Rank 1	Rank 2	Rank 3	Rank 4	Rank 5	Rank 6	Rank 7
1	0.247825	0.190020	0.176455	0.141435	0.085875	0.062260	0.096130
2	0.024185	0.113125	0.245550	0.295265	0.165180	0.102740	0.053955
3	0.051385	0.075740	0.143330	0.162195	0.301295	0.143850	0.122205
4	0.006370	0.018115	0.053935	0.112500	0.225815	0.356550	0.226715
5	0.000115	0.000865	0.008310	0.035275	0.146635	0.312755	0.496045
6	0.566100	0.259910	0.106530	0.042330	0.015945	0.006380	0.002805
7	0.104020	0.342225	0.265890	0.211000	0.059255	0.015465	0.002145

1. tebentafusp; 2. ICIs; 3. targeted therapy combined with chemotherapy; 4. targeted therapy alone; 5. chemotherapy; 6. LDT-ICIS; 7.LDT.

#### Overall survival

**Figure 4:**
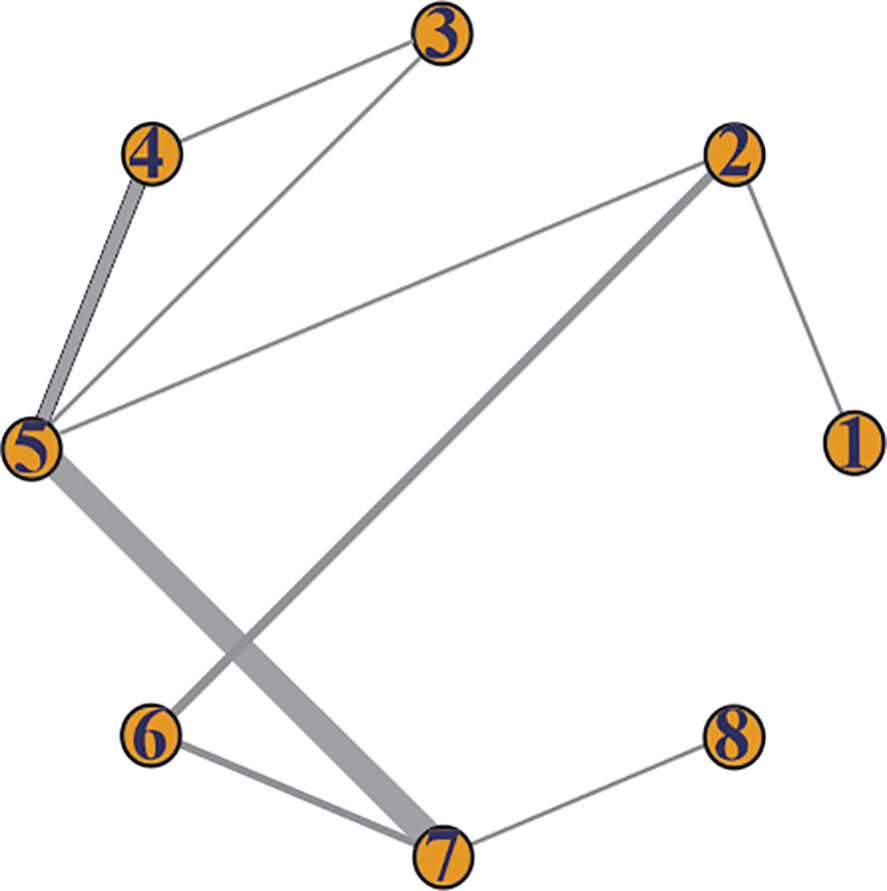
Network diagram of OS. 1. tebentafusp; 2. ICIs; 3. targeted therapy plus chemotherapy; 4. targeted therapy alone; 5. chemotherapy; 6. LDT-ICIs; 7. LDT; 8. LDT plus chemotherapy.

Overall survival information was reported in all 17 studies, including 8 different treatment modalities. The network diagram was shown in **Figure 4**.

**Figure 5:**
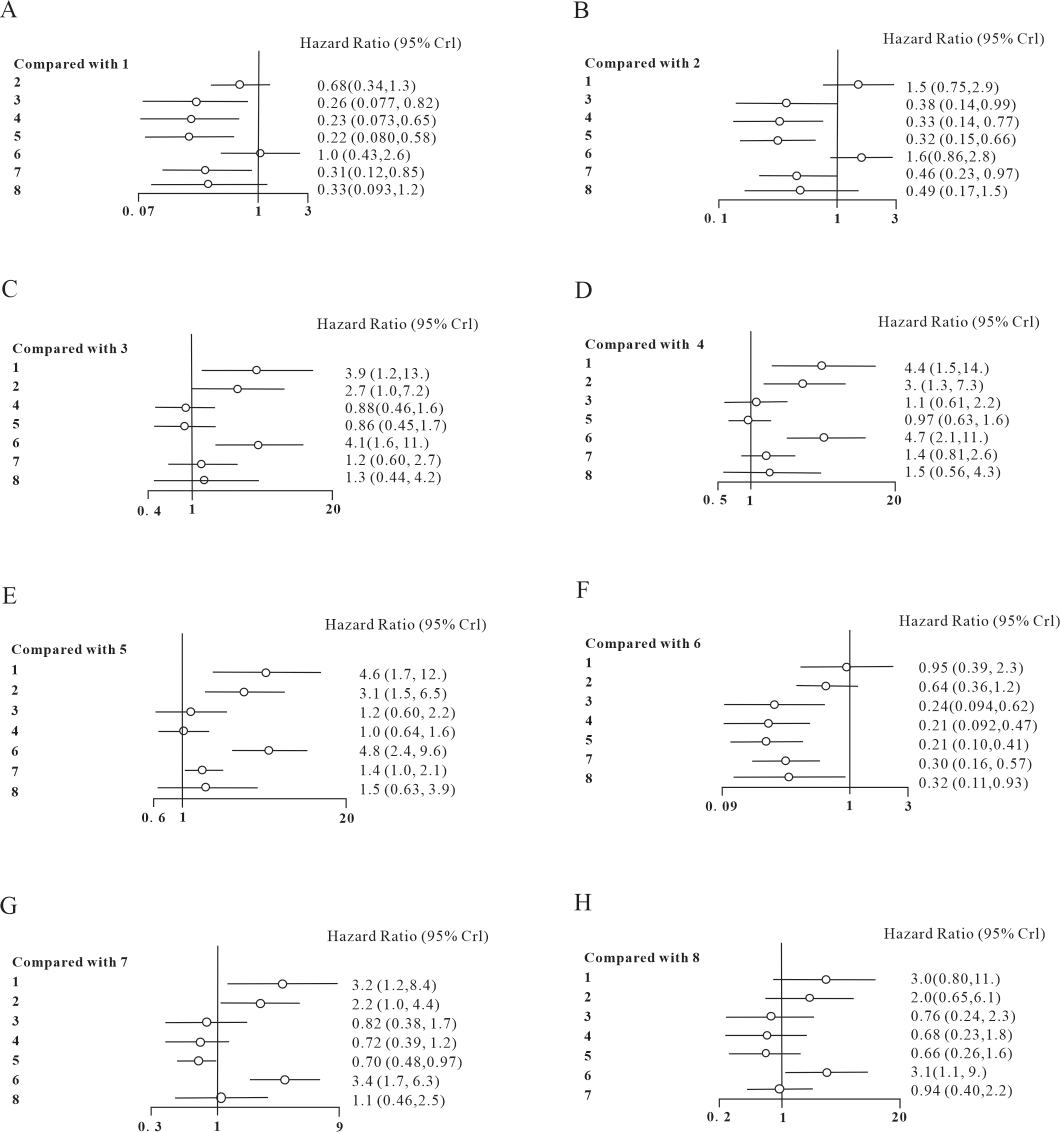
Forest plot of comparison of OS. 1. tebentafusp; 2. ICIs; 3. targeted therapy plus chemotherapy; 4. targeted therapy alone; 5. chemotherapy; 6. LDT-ICIs; 7. LDT; 8. LDT plus chemotherapy. **(A)** compared with tebentafusp; **(B)** compared with ICIs; **(C)** compared with targeted therapy combined with chemotherapy; **(D)** compared with targeted therapy alone; **(E)** compared with chemotherapy; **(F)** compared with LDT-ICIs; **(G)** compare with LDT; **(H)** compared with LDT plus chemotherapy.

As illustrated in the forest plot for overall survival (**Figure 5**), the ICIs demonstrated superior efficacy compared to the other four therapies in enhancing OS. These included targeted therapy plus chemotherapy (HR 0.38, 95% CI:[0.14,0.99]), targeted therapy alone (HR 0.33, 95% CI:[0.14, 0.77]), chemotherapy (HR 0.32, 95% CI:[0.15, 0.66]), LDT (HR 0.46, 95% CI:[0.23, 0.97]). However, the efficacy of other therapies such as tebentafusp (HR 1.5,95% CI: [0.75, 2.9]), LDT-ICIS (HR 1.6,95% CI: [0.86, 2.8]), and LDT plus chemotherapy (HR 0.49, 95% CI:[0.17, 1.5]) did not show statistically significant differences from the ICIs. Similarly, the therapy of tebentafusp were also better than the other four therapies, which were targeted therapy plus chemotherapy (HR 0.26, 95% CI:[0.077,0.82]), targeted therapy alone (HR 0.23,95% CI: [0.073, 0.65]), chemotherapy (HR 0.22, 95% CI:[0.08, 0.58]), and LDT (HR 0.31, 95% CI:[0.12, 0.85]). Furthermore, the LDT-ICIs was superior to targeted therapy plus chemotherapy (HR 0.24, 95% CI:[0.094,0.62]), targeted therapy alone (HR 0.21, 95% CI:[0.092, 0.47]), chemotherapy (HR 0.21, 95% CI:[0.10, 0.41]), LDT (HR 0.30, 95% CI:[0.16, 0.57]),and LDT plus chemotherapy (HR 0.32, 95% CI:[0.11, 0.93]). The tebentafusp (HR 3.0, 95% CI:[0.8,11]) and ICIs (HR 2.0, 95% CI:[0.65, 6.1]) did not show statistically significant difference from the LDT plus chemotherapy instead. The liver-directed therapy was slightly better than chemotherapy (HR 1.4, 95% CI:[1.0, 2.1]).

In terms of overall survival improvement (**Table 3**), the LDT-ICIs is most likely to secure the top ranking. It is followed by tebentafusp, ICIs, and LDT plus chemotherapy. The liver-directed therapy, the targeted therapy plus chemotherapy, the chemotherapy, and the targeted therapy are ranked at the bottom.

**Table 3:** Ranking chart of OS.

Therapeutic modality	Rank 1	Rank 2	Rank 3	Rank 4	Rank 5	Rank 6	Rank 7	Rank 8
1	0.442975	0.441700	0.084595	0.018180	0.006360	0.002590	0.001960	0.001640
2	0.008555	0.124420	0.763265	0.083315	0.014000	0.004075	0.001590	0.000780
3	0.001220	0.005265	0.013795	0.152980	0.179775	0.271790	0.171545	0.203630
4	0.000065	0.000745	0.002140	0.032380	0.092355	0.231775	0.334705	0.305835
5	0.000005	0.000035	0.000190	0.002520	0.038150	0.220330	0.374695	0.364075
6	0.535635	0.395195	0.064715	0.003870	0.000405	0.000100	0.000055	0.000025
7	0.000075	0.002335	0.010780	0.300210	0.477390	0.163800	0.040935	0.004475
8	0.011470	0.030305	0.060520	0.406545	0.191565	0.105540	0.074515	0.119540

1.tebentafusp; 2.ICIs; 3.targeted therapy plu**1s** chemotherapy; 4.targeted therapy alone; 5.chemotherapy; 6.LDT-ICIs; 7.LDT; 8.LDT plu**1s** chemotherapy.

## Discussion

The primary objective of this study was to evaluate and compare the effectiveness of different therapeutic modalities in patients with metastatic uveal melanoma. So far, treatment patterns of patients with metastatic UM are extremely heterogeneous (44). Although the NCCN guideline currently recommends tebentafusp as preferred first-line treatment in HLA-A*02:01-positive patients with metastatic diseases, it does not address outcomes in HLA-A*02:01-negative patients or resource-limited settings lacking tebentafusp. Therefore, to ensure broad clinical applicability, our study population was limited to patients with hepatic metastases from uveal melanoma, with no exclusion based on HLA-A*02:01 genotype. We also reviewed other immunotherapies beyond immune-mobilizing monoclonal T-cell receptors and ICIs, such as tumor-infiltrating lymphocyte therapy (45), tumor vaccine (46), oncolytic virotherapy (47). These were excluded from the analysis because they were only investigated in single-arm phase I trials or are currently being evaluated in ongoing comparative studies that have not yet been completed (e.g., NCT06581406).

The results of our analysis revealed that the LDT-ICIs had the highest efficacy in terms of improving patients’ PFS. Our comparative efficacy analysis demonstrated that both liver-directed therapy alone and in combination with ICIs were significantly superior to chemotherapy. However, neither approach showed a statistically significant advantage over tebentafusp or ICIs therapy. In the relative efficacy ranking, the top three therapeutic strategies were LDT-ICIs, LDT, and ICIs, while tebentafusp ranking last. This finding showed discordance with both the relative efficacy analysis and the clinical trial result reported by Hassel et al. (10) We attribute this discordance partly to the limited evidence base for tebentafusp in our analysis—only one randomized trial was included, resulting in low statistical weight and potentially unreliable ranking. Researchers have also noted that the PFS of tebentafusp and ICIs was low when compared to the magnitude of survival benefit (8). Cumulatively, PFS served as an indicator to evaluate the degree of tumor growth controlled by treatment, which was determined by radiographic progression according to RECIST v1.1. However, RECIST criteria may lack sensitivity in capturing meaningful clinical benefits of systemic therapy for patients with LMUM. Across studies, objective response rates based on RECIST criteria, defined as the total proportion of complete and partial responses, were consistently low. Nevertheless, heterogeneous survival benefits were observed among these patient cohorts. The observed survival extension cannot be accounted for by such a limited objective response rate. The observed dissociation between objective response rates (ORR) and long-term survival outcomes suggests that RECIST criteria primarily reflect unidimensional tumor size reduction, while failing to incorporate temporal dynamics of treatment response. Immunotherapy, in particular, often requires longer follow-up than conventional treatments due to its carryover effects. This means median PFS may not fully reflect the clinical benefits of immunotherapy. In metastatic cancer, some researchers argued that tumor growth rate, rather than tumor volume, could be a more suitable metric for assessing the efficacy of immune-based interventions (48). Thus, PFS is poor for LMUM and probably does not represent the ideal outcome for the comparison of distinct treatments. The results for radiographic response and PFS underestimated the overall survival benefit with tebentafusp.

Notably, our findings demonstrated that liver-directed therapy combined with ICIs was also the most effective approach for improving OS. LDT-ICIs, tebentafusp, and ICIs all showed significantly better OS outcomes compared to liver-directed therapy alone, chemotherapy, targeted therapy, or combinations of chemotherapy with kinase inhibitors. These results remained consistent with the relative effect ranking observed in our analysis. In a pivotal high-quality phase III randomized controlled trial (10)and its corresponding 3-year follow-up publication (43), treatment with tebentafusp resulted in significantly longer overall survival than the investigator’s choice of treatment with single-agent immune checkpoint inhibitors or chemotherapy drugs (pembrolizumab, ipilimumab, or dacarbazine).

Melanoma is generally refractory to conventional chemotherapy. Unlike cutaneous melanoma, uveal melanoma exhibits a low tumor mutational burden, which may contribute to its reduced responsiveness to immune checkpoint inhibitors (49). Current evidence from a single-arm study of combined immune checkpoint inhibitors in metastatic uveal melanoma demonstrated a 12-month overall survival (OS) rate of 51.9% (95% CI 38.3 to 65.5), with median OS of 12.7 months and PFS of 3.0 months (9). However, no direct comparative data exist between this approach and tebentafusp. Therefore, Piulats et al. (50)conducted a propensity score-weighted analysis comparing tebentafusp with dual immune checkpoint inhibitors using available pooled data. The inverse probability of treatment weighting (IPTW) analysis showed that OS favored tebentafusp (HR 0.52 [95% CI 0.35-0.78]); 1-year OS was 73% for tebentafusp versus 50% for dual ICIs. Sensitivity analyses showed consistent superior OS for tebentafusp with all IPTW HRs 0.61. Nevertheless, the current first-line recommendation for tebentafusp in HLA-A*02:01-positive metastatic uveal melanoma leaves unmet needs for HLA-ineligible patients and healthcare systems with limited drug availability.

Metastatic uveal melanoma demonstrates a high predilection for hepatic metastases and thrives within the liver’s immunosuppressive microenvironment—a combination that has prompted extensive investigation of local therapeutic strategies for hepatic disease (22). Surgical resection was associated with improved overall survival in selected patients, though eligibility remains limited by multifocal or bilobar disease (20). Liver-directed therapies such as radiofrequency ablation, radiotherapy, chemoembolization, immunoembolization, radioembolization, SIRT, IHP and PHP, was employed to treat metastatic liver disease. Our analysis indicated that liver-directed monotherapy was significantly more effective than chemotherapy, with a superior relative-effect ranking compared to both targeted therapy and its combination with chemotherapy. This observation is consistent with the known limited responsiveness of metastatic melanoma to systemic treatments (51). Analysis of liver-directed therapy combined with systemic treatments revealed that the combination of chemotherapy with liver-directed therapy provided only a modest benefit over liver-directed therapy alone (a single position higher in the efficacy ranking). By contrast, the LDT-ICIs was associated with significantly improved outcomes compared with liver-directed monotherapy (HR 0.30, 95% CI:[0.16, 0.57]). A retrospective comparative analysis evaluated dual immune checkpoint inhibitors combined with SIRT versus SIRT alone and demonstrated a significant prolonged OS in the combination group with ICIs (46.6 vs. 11.8 months, p = 0.039) (32). Emerging evidence indicated that Y90 Radioembolization combined with immunotherapy yields significantly prolonged overall survival compared to TARE alone (20.6 versus 9.5 months, P = 0.014) (36). Taken together, these data supports that radiotherapy and immunotherapy synergize to enhance the efficacy of the treatments. While our relative effect estimates did not demonstrate a significant advantage of LDT-ICIs over ICIs alone (HR 1.6, 95% CI 0.86–2.8), existing data indicated a significant prolongation of median OS in patients receiving combination therapy versus ICIs monotherapy (20.1 vs. 13.8 months; p=0.0016) (38). Another retrospective study showed that the LDT-ICIs conferred a significant survival advantage compared with ICIs monotherapy (22.5 months vs. 11.4 months, p = 0.036) (39). These findings may explain the higher ranking of LDT-ICIs combination therapy in current evidence. However, given that only two comparative studies exist—with limited weight due to potential inter-site variability in protocol adherence and the retrospective nature of the analyses—further prospective comparative studies are warranted to confirm the superiority of LDT-ICIs. There is now robust evidence that LDT-ICIs combination therapy significantly improves therapeutic response rates. Since liver-directed therapies lead to an increased release of tumor antigens and, thus, to a higher tumor mutational burden (52), the simultaneous use of immunotherapy could result in a greater immune response in mUM. Through their complementary mechanisms, the combination of liver-directed therapy and immunotherapy presents a clinically promising strategy for metastatic uveal melanoma, without being constrained by the patient’s HLA-A*02:01 subtype. In HLA-A*02:01-positive patients, we found insufficient evidence to conclude that LDT-ICIs prolongs OS compared to tebentafusp, despite the combination therapy ranked higher in relative effect measures. Key limitations include: 1) only one available tebentafusp study, exclusively involving HLA-A*02:01-positive patients; 2) unreported HLA-A*02:01 status in other relevant studies. These evidence gaps highlight the need for direct comparative studies to establish whether the combination approach is truly superior to tebentafusp for HLA-A*02:01-positive liver metastatic uveal melanoma patients. Ongoing clinical trials are assessing multiple liver-directed modalities SIRT (NCT02913417), immunoembolization (NCT03472586), IHP (NCT04463368) combined with immunotherapy. Preliminary data from a PHP-plus-ICIs regimen has shown encouraging response rates (53).

The targeted therapies evaluated in the included studies comprised selumetinib, cabozantinib, and sunitinib. Selumetinib, a MEK inhibitor, is associated with development of resistance due to adaptive GPCR-mediated YAP activation and AKT signaling pathways (54). Cabozantinib and sunitinib are tyrosine kinase inhibitors; however, their efficacy is limited in UM owing to the low prevalence of c-KIT mutations (55). Consequently, in our analysis, targeted therapy demonstrated outcomes comparable to those of chemotherapy in both PFS and OS.

Meanwhile, current therapeutic development for mUM focuses on novel targeted agents and rational combination strategies. Binimetinib, a MEK inhibitor, is being evaluated in combination with the HDAC inhibitor belinostat in a phase II trial (NCT05170334), and with darovasertib in a phase I/II trial (NCT03947385). Darovasertib is also under investigation in combination with crizotinib via a phase I/II basket trial (NCT03947385), which includes both pretreated and treatment-naïve mUM patients. Promisingly, this combination has demonstrated antitumor activity irrespective of HLA-A*02:01 status (56). Based on these data, a large randomized phase II/III trial (NCT05987332) is now comparing darovasertib plus crizotinib versus investigator’s choice as first-line therapy in HLA-A*02:01-negative mUM patients. The PI3K/AKT pathway is also constitutively active in most UMs (57). IOA-244 (roginolisib), which inhibits PI3Kδ-dependent signaling in tumor cells and regulatory T cells (58), is being tested against investigator’s choice in a phase II trial involving mUM patients (NCT06717126). In the phase II PEMDAC trial (NCT02697630), combination therapy with the HDAC inhibitor entinostat and pembrolizumab resulted in an objective response rate of 14%, a median progression-free survival of 2.1 months, and a median overall survival of 13.4 months in patients with metastatic uveal melanoma (mUM), alongside a manageable toxicity profile (59). Separately, a phase II trial evaluating vorinostat, another HDAC inhibitor, in mUM patients (NCT01587352) is ongoing, with results pending. We will continue to monitor outcomes from these studies, particularly those of darovasertib plus crizotinib in HLA-A*02:01–negative patients. These results may substantially alter the treatment paradigm and potentially challenge the established role of tebentafusp.

Regarding objective response rate (ORR), the inability to construct a network meta-analysis must be acknowledged, as this endpoint was not consistently reported across studies. Based on available data, liver-directed therapy unequivocally demonstrated the highest ORR for hepatic metastases—showing clear superiority over chemotherapy in direct comparisons. Furthermore, the combination of liver-directed therapy with ICIs has shown enhanced efficacy over either modality alone in several studies. However, current evidence did not permit reliable estimation of OR to determine the comparative efficacy of tebentafusp, ICIs, targeted agents, and chemotherapy based on ORR.

In terms of safety, tebentafusp was associated with grade 3 or 4 treatment-related adverse events in approximately 47% of cases, predominantly manifesting as cutaneous rash, followed by pyrexia and pruritus - representing a higher incidence than observed with ICIs. Targeted therapy demonstrated a greater burden of treatment-related toxicities compared to chemotherapy, with fatigue and transaminase elevation (increased AST/ALT) being most frequently reported. The safety profile of liver-directed therapies varied relative to chemotherapy, with some studies showing higher and others lower. The combination of liver-directed therapy with immunotherapy exhibited increased adverse events compared to ICIs monotherapy. Importantly, currently available data suggested these adverse events remain clinically manageable in most cases.

This study is subject to several limitations. Despite its novelty, our analysis incorporated several retrospective studies, particularly those investigating liver-directed therapy in combination with other treatment modalities. Due to the different timing of the various therapies, the population of this study is heterogeneous and prospective studies are warranted to confirm that the additional use of LDT increases the efficacy of systemic treatments. Moreover, direct comparisons between different liver-directed therapy modalities remain challenging to establish due to significant variations in patient selection criteria and treatment protocols across studies. In addition, patients receiving LDT combined with ICI may have had a lower baseline tumor burden compared with those receiving other treatments such as targeted therapy, as LDT trials tend to enroll patients with less extensive disease. This potential selection bias, along with the lack of adequate baseline tumor burden data in most included studies, may have influenced the comparative outcomes. Furthermore, the prognostic role of HLA phenotypes in uveal melanoma patients receiving immunotherapy remains unclear based on current evidence, and we were unable to account for this factor due to insufficient reporting across studies. While these studies provide valuable real-world insights, their inclusion necessitates careful interpretation due to the inherent limitations of retrospective designs, including potential selection bias and heterogeneous treatment protocols. Second, our analysis did not account for baseline lactate dehydrogenase (LDH) levels or the presence of extrahepatic metastases - both established prognostic factors for OS and PFS (6). The original studies lacked consistent stratification by these variables, potentially introducing confounding bias.

Finally, emerging immunotherapies and targeted therapies are often evaluated in single-arm trials, which limits their inclusion in network meta-analyses. Additionally, many ongoing clinical trials have yet to report outcomes. Combination therapies represent a promising area for further investigation and should be a focus of future research and inclusion in subsequent analyses.

## Conclusion

Our analysis summarizes current evidence regarding the efficacy of diverse therapeutic modalities for liver metastatic uveal melanoma. It demonstrates that the combination of LDT and ICIs confers superior PFS and OS benefits compared with alternative therapies, including tebentafusp and ICIs monotherapy, without considering HLA-A*02:01 subtype particularly. The LDT-ICIs also demonstrated acceptable tolerability, with manageable treatment-related adverse effects. These results underscore the therapeutic promise of combining local and systemic strategies to leverage synergistic effects within the immunosuppressive hepatic microenvironment.

Future efforts should focus on validating LDT-ICIs in prospective randomized trials, standardizing treatment protocols across centers, and exploring novel targeted and immunotherapeutic agents—especially in both HLA-A*02:01-positive and negative cohorts. Until such data are available, LDT-ICIs represents a compelling treatment strategy for mUM patients with hepatic involvement, including those ineligible for or without access to tebentafusp.

## Data Availability

The original contributions presented in the study are included in the article/**Supplementary Material**. Further inquiries can be directed to the corresponding author.
