# Supplementary material for: Comparative efficacy of therapeutic modalities for metastatic uveal melanoma: a systemic review and network meta-analysis
Source: Front Oncol. 2026 May 20;16:1811719. doi: 10.3389/fonc.2026.1811719 (PMC13229724; doi:10.3389/fonc.2026.1811719)
Supplement: Supplementary file 1 [file Table1.docx]

**Supplementary material :**

**Table S1. Search strategy**

| Database | Pubmed | Embase | Cochrane |
| --- | --- | --- | --- |
| Time Span | January 2020 – March 2026 | | |
| Search Query | (metastatic uveal melanoma[Title/Abstract] OR liver metastatic uveal melanoma[Title/Abstract]) AND (immunotherapy[Title/Abstract] OR tebentafusp[Title/Abstract] OR immune checkpoint inhibitors[Title/Abstract] OR immune checkpoint blockade[Title/Abstract] OR immunological checkpoint inhibitor[Title/Abstract] OR targeted therapy[Title/Abstract] OR molecular targeted therapy[Title/Abstract] OR leukemia targeted treatment[Title/Abstract] OR kinase inhibitor[Title/Abstract] OR chemotherapy[Title/Abstract] OR liver-directed therapy[Title/Abstract] OR liver-specific therapy[Title/Abstract] OR surgical resection[Title/Abstract] OR radiofrequency ablation[Title/Abstract] OR transarterial chemoembolization[Title/Abstract] OR immunoembolization[Title/Abstract] OR selective internal radiotherapy[Title/Abstract] OR isolated hepatic perfusion[Title/Abstract] OR percutaneous hepatic perfusion[Title/Abstract] OR locoregional treatment[Title/Abstract] OR intra-hepatic treatment[Title/Abstract]) | #3 #1 AND #2  #2 'tebentafusp:ab,ti OR 'immunotherapy':ab,ti OR 'immune checkpoint inhibitors':ab,ti OR 'immune checkpoint blockade':ab,ti OR 'immunological checkpoint inhibitor':ab,ti OR 'targeted therapy':ab,ti OR 'molecular targeted therapy':ab,ti OR 'leukemia targeted treatment':ab,ti OR 'kinase inhibitor':ab,ti OR chemotherapy:ab,ti OR 'liver-directed therapy':ab,ti OR 'liver-specific therapy':ab,ti OR 'surgical resection':ab,ti OR 'radiofrequency ablation':ab,ti OR 'transarterial chemoembolization':ab,ti OR immunoembolization:ab,ti OR 'selective internal radiotherapy':ab,ti OR 'isolated hepatic perfusion':ab,ti OR 'percutaneous hepatic perfusion':ab,ti OR 'locoregional treatment':ab,ti OR 'intra-hepatic treatment':ab,ti  #1 'metastatic uveal melanoma':ab,ti OR 'liver metastatic uveal melanoma':ab,ti | #1 (metastatic uveal melanoma or liver metastatic uveal melanoma):ti,ab,kw  #2 (tebentafusp or immunotherapy or immune checkpoint inhibitors or immune checkpoint blockade or immunological checkpoint inhibitor or targeted therapy or molecular targeted therapy or leukemia targeted treatment or kinase inhibitor or chemotherapy or liver-directed therapy or liver-specific therapy or surgical resection or radiofrequency ablation or transarterial chemoembolization or immunoembolization or selective internal radiotherapy or isolated hepatic perfusion or percutaneous hepatic perfusion or locoregional treatment or intra-hepatic treatment):ti,ab,kw  #3 #1 AND #2 |
